# Validating the Michigan Hand Outcomes Questionnaire in patients with rheumatoid arthritis using Rasch analysis

**DOI:** 10.1371/journal.pone.0254984

**Published:** 2021-07-22

**Authors:** Mayank Jayaram, Chang Wang, Lu Wang, Kevin C. Chung

**Affiliations:** 1 University of Michigan Medical School, Ann Arbor, Michigan, United States of America; 2 University of Michigan School of Public Health, Ann Arbor, Michigan, United States of America; 3 Section of Plastic Surgery, Michigan Medicine, Ann Arbor, Michigan, United States of America; University of Copenhagen, DENMARK

## Abstract

**Introduction:**

The Michigan Hand Outcomes Questionnaire (MHQ) is a patient-reported outcome measure previously validated in patients with rheumatoid arthritis (RA) using classical test theory. Rasch analysis is a more rigorous method of questionnaire validation that has not been used to test the psychometric properties of the MHQ in patients with RA. The objective of this study is to evaluate the validity and reliability of the MHQ for measuring outcomes in patients with RA with metacarpophalangeal joint deformities.

**Methods:**

We performed a Rasch analysis using baseline data from the Silicone Arthroplasty in Rheumatoid Arthritis (SARA) prospective cohort study. All domains were tested for threshold ordering, item fit, targeting, differential-item functioning, unidimensionality, and internal consistency.

**Results:**

The Function and Work domains showed excellent fit to the Rasch model. After making adjustments, the Pain, Activities of Daily Living (ADL) and Satisfaction domains also fulfilled all Rasch model criteria. The Aesthetics domain met the majority of Rasch criteria, but could not be tested for unidimensionality.

**Conclusions:**

After collapsing disordered thresholds and removing misfitting items, the MHQ demonstrated reliability and validity for assessing outcomes in patients with RA with metacarpophalangeal joint deformities. These results suggest that interpreting individual domain scores may provide more insight into a patient’s condition rather than analyzing an overall MHQ summary score. However, more Rasch analyses are needed in other RA populations before making adjustments to the MHQ.

## Introduction

Rheumatoid arthritis (RA) is a progressive autoimmune disease that primarily affects joints in the hands, wrists, and knees, and can lead to severe pain and swelling in the affected region. Nearly 1.3 million adults in the United States are living with RA, and approximately 70% will experience some impairment to hand performance as a result of the disease [1, 2]. RA often leads to disfiguration at the metacarpophalangeal (MCP) joint, resulting in ulnar deviation and reduced satisfaction with the appearance of the hand [3]. Silicone metacarpophalangeal joint arthroplasty (SMPA) can be used to treat MCP joint deformities in patients with RA; however, there is no consensus on the long-term benefits of this procedure [4]. As a result, it is necessary to develop and validate reliable instruments in the RA population that incorporate the patient’s perspective into assessment of treatment outcomes [5].

Patient-reported outcome measures (PROMs) are assessment tools used in clinical research to gain insight into how a patient perceives outcomes following treatment. PROMs can be helpful in hand/wrist conditions to inform investigators about how quality of life measures such as perceived pain after injury can influence outcomes [6]. The Michigan Hand Outcomes Questionnaire (MHQ) is a 37-item PROM frequently used to measure outcomes in musculoskeletal disorders involving the hand/wrist. Since its development, the MHQ has been used in several multinational clinical trials to study conditions such as RA, distal radius fractures, and finger amputations [7–9]. The MHQ has been translated into 12 languages and validated in numerous upper extremity musculoskeletal conditions [10–14].

Previous methods used to validate the MHQ for RA relied on classical test theory (CTT). However, Rasch analysis provides an alternative method of questionnaire validation that more comprehensively tests the psychometric properties of a questionnaire [15]. Specifically, Rasch analysis investigates each item for biases, redundancy, and ambiguous wording [16]. Results from Rasch analyses can be used to eliminate poorly functioning items in the questionnaire and optimize a PROM for the condition of interest.

The purpose of this study is to use Rasch analysis to rigorously test the psychometric properties of the MHQ in a RA study population. Results from this study can strengthen the credibility of the MHQ as a valid and reliable outcome measure for individuals with RA-related hand deformities.

## Materials and methods

### MHQ

An expert panel comprised of patients with hand disorders, hand therapists, and hand surgeons were involved in the development of the MHQ [11]. These individuals used previously validated hand questionnaires to categorize a preliminary list of 100 items into 6 domains (Function, Activities of Daily Living (ADLs), Work, Pain, Aesthetics, Satisfaction) that each measure a unique construct related to hand performance. Two psychometricians evaluated the initial 100 items in the MHQ for item redundancy and ambiguous wording and then factor analysis was performed to further optimize the number of items in each scale. Following factor analysis, the MHQ was pilot tested in a cohort of 200 patients before the final version was developed. The finalized MHQ contains 37 items with 5 response options per question. Each of the 6 domains is scored on a scale between 0 and 100. For the pain domain, lower scores represent less pain, and for all other domains, higher scores indicate better hand health.

### Study sample

For this analysis, we used data from the Silicone Arthroplasty in Rheumatoid Arthritis (SARA) prospective cohort study. SARA is one of the largest studies to collect information on patients with rheumatoid arthritis with severe deformities at the MCP joint [17]. Patients were recruited from two sites in the United States and one site in the United Kingdom. Study participants were divided into two cohorts: surgical treatment with SMPA or medical management without surgery (non-SMPA). The MHQ was used to measure patient-reported outcomes at baseline and at several intervals following treatment in the follow-up period. Rasch analysis was performed using baseline MHQ scores, and all participants were combined into one cohort for analysis.

### Rasch analysis

A key requirement for Rasch analysis is unidimensionality, so the entire methodology was applied to each domain. The methodology for this analysis is based on Tennant and Conaghan’s criteria for Rasch analysis [18]. This method has been previously used to validate the MHQ [19].

### Model choice

The likelihood ratio test was performed to determine which Rasch model to use. The rating scale model (RSM) assumes each response option in a category are equidistant. The RSM is nested within the partial credit model (PCM), which assumes the threshold distance between response options are not equally spaced. A non-significant likelihood ratio indicates equidistant response options and fulfils the assumptions of the RSM allowing us to simplify the model and facilitate interpretation. A significant likelihood ratio indicates thresholds between response options are not equally spaced and the partial credit model (PCM) should be used [18].

### Threshold ordering

A threshold is observed when an individual has an equal chance of selecting between two response options in a questionnaire. In the MHQ, a threshold would occur if an individual is equally likely to select “very good” or “good” to describe his/her finger mobility. However, if individuals have difficulty discriminating between these response options, disordered thresholds could result. For example, disordered thresholds would occur if individuals with excellent hand function select “good” to describe their finger mobility, whereas individuals with worse hand function select “very good.” In this scenario the ordering of response options by severity of hand dysfunction is not in congruence with the expectations of the questionnaire, and individuals will not be accurately sorted by hand ability. Disordered thresholds are the result of ambiguous wording or too many response options for the item and are addressed by collapsing categories to preserve the latent structure of the MHQ [20].

### Item fit

Item fit measures how well observed data meets the expectations of the Rasch model. If responses to certain items are different from model expectations, the item misfits the Rasch model. Item fit was assessed at the individual item and overall domain levels. Individual item fit was assessed by deriving chi-square (X^2^) statistics from the residual sum of squares [21]. Significant p-values (p <0.05) following Bonferroni adjustment indicate poor fit to the Rasch model [18]. Bonferroni adjustment is a method used to test item fit for a group of items within a specific domain. For a group of items with size k, Bonferroni adjustment compares the minimal p-value with 0.05/k. Therefore, in our case with 5 items, we compare to a significance level of 0.01.

Overall domain fit was assessed using the information-weighted (infit) and outlier-sensitive (outfit) mean square statistics (MNSQ). If the items perfectly fit the Rasch model, they will have MNSQ statistics approximately equal to 1 [22]. Infit MNSQ scores <1 indicate overfit or redundancy, and outfit MNSQ scores >1 indicate underfit and large deviations between observed and expected behavior to the Rasch model [22]. MNSQ scores between 0.6 and 1.4 are acceptable for overall fit to the Rasch model [23]. An overall test of model fit for each domain was also performed using Andersen’s conditional likelihood ratio with a significance of p <0.05 [24].

### Targeting

All items in the MHQ were stratified by item difficulty with easier questions located at one end of the continuum and difficult questions located at the other end. Similarly, all people were separated across the same continuum based on person-ability. A well targeted measure is one that has mean item difficulties at a similar location to average person-ability. Poorly targeted measures have items that are too difficult or too simple to accurately evaluate the study population. Targeting will be assessed by visually inspecting the person-item map to ensure each domain captures the broad range of ability levels of the study population [25].

### Differential-item functioning

Differential-item functioning (DIF) occurs when individuals respond differently to an item based on characteristics such as age, gender, socioeconomic status, etc. DIF can be classified as uniform or non-uniform. Uniform DIF occurs when the differences in responses are consistent across the characteristic being measured and is addressed by splitting the groups and independently calibrating each subgroup to the Rasch model [26]. Non-uniform DIF occurs when differences in response options are inconsistent across the trait being measured and implies an inherent issue with the item that may be causing the abnormal response pattern. Non-uniform DIF is addressed by removing the item from the Rasch model [27].

For this analysis, DIF was tested for dominant hand, education level, and location (US or UK). Three models were generated. Model 1 assumes no DIF exists, model 2 assumes uniform DIF exists, and model 3 assumes non-uniform DIF exists. If there is a significant difference between models 1 and 2, the item exhibits uniform DIF, and if there is a significant difference between models 2 and 3, the item has non-uniform DIF and will be removed from analysis [28].

### Unidimensionality

Each domain was analyzed for unidimensionality using the Martin-Lӧf test. The Martin-Lӧf test is used to evaluate if all the items in a domain are related by a single factor indicating unidimensionality. A significant result (p<0.05) indicates multidimensionality [29]. Response dependency is another method to test for unidimensionality and occurs when a participant’s answer to one item influences their response to another item in the questionnaire, thereby invalidating local independence. If the correlation between two items is > 0.3 from the average residual correlation for all items in the domain, the items demonstrate response dependency [30].

### Internal consistency

Internal consistency measures reliability and was calculated using Cronbach’s α. A Cronbach’s α >0.70 indicates high reliability, while a Cronbach’s α >0.90 indicates high internal consistency with redundancy [31]. All the statistical analyses were performed using R version 4.0.2 with a significance level of p-value<0.05.

## Results

The final study population included 162 participants from SARA who completed the MHQ at enrollment. The majority of enrolled participants were white (89%), female (73%), and had an income of ≤$50,000/year (72%) (Table 1). The likelihood ratio was significant for all domains except Function. Therefore, the RSM was used for Function and the PCM was used for all other domains. The conditional likelihood ratio for all domains was p>0.05 indicating good model fit for each domain (Table 2).

**Table 1:** Demographic characteristics for the Silicone Arthroplasty in Rheumatoid Arthritis Cohort.

Total Participants (n)	162
**Age, mean (SD)**	**61 (10)**
**Male, No. (%)**	**44 (27)**
**Race, White, No. (%)^a^**	**137 (89)**
**Education, ≤ High School Degree, No. (%)^a^**	**73 (47)**
**Income, ≤ $50,000, No. (%)^a^**	**107 (72)**

^a^Eight (5%) participants are missing race and education data and 13 (8%) are missing income data.

**Table 2:** Andersen’s conditional likelihood ratio (CLR) for each domain.

Domain	Chi Square (X^2^)	P-value	Degrees of Freedom
**Function**	8.2	0.32	7
**ADL**	45.3	0.46	45
**Work**	39.9	0.05	19
**Pain**	11.6	0.90	19
**Aesthetics**	5.6	0.47	6
**Satisfaction**	9.5	0.89	16

### Function

**Fig 1:**
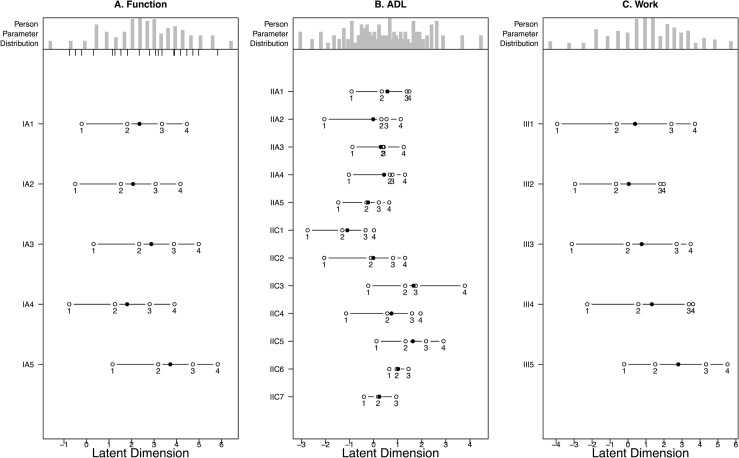
Person-Item Map and Threshold Ordering for Function (1a, left), ADLs after collapsing disordered thresholds (1b, middle), and Work (1c, right).

No disordered thresholds were observed (Fig 1A) and all items fit well to the Rasch model (Table 3). Function was well targeted and no DIF was observed for dominant hand, education level, or location. Function had high internal consistency (Cronbach’s α = 0.87), was unidimensional according to the Martin-Lӧf test (p = 0.99), and demonstrated no response dependency (Table 4). Overall, Function fit the Rasch model extremely well.

**Table 3:** Fit to the Rasch model.

**Function Domain**							
**Item**	**Item Statement**	**MNSQ Infit**^a^	**MNSQ Outfit**^b^	**Chi Square (X**^**2**^**)**	**P-value**	**DOF**^c^	**DIF**^d^ **or Misfit?**
1	How well did your hand work?	0.515	0.506	80.918	1	161	
2	How well did your fingers move?	0.612	0.619	99.074	1	161	
3	How well did your wrist move?	1.263	1.261	201.796	0.012	161	
4	How was the strength?	0.761	0.745	119.221	0.992	161	
5	How was the sensation (feeling)?	0.960	1.077	172.392	0.221	161	
**Activities of Daily Living Domain**							
1	How difficult was it for you to turn a door knob?	0.768	0.692	107.900	0.999	155	non-uniform DIF dominant hand
2	How difficult was it for you to pick up a coin?	0.976	0.999	155.878	0.465	155	non-uniform DIF dominant hand
3	How difficult was it for you to hold a glass of water?	1.061	1.018	158.820	0.400	155	non-uniform DIF dominant hand
4	How difficult was it for you to turn a key in a lock?	0.827	0.747	116.521	0.991	155	non-uniform DIF dominant hand
5	How difficult was it for you to hold a frying pan?	0.878	0.893	139.342	0.811	155	
6	How difficult was it for you to open a jar?	1.355	1.119	174.536	0.135	155	
7	How difficult was it for you to button a shirt/blouse?	0.952	0.882	137.615	0.839	155	
8	How difficult was it for you to eat with a knife/fork?	0.902	0.869	135.609	0.867	155	
9	How difficult was it for you to carry a grocery bag?	1.062	1.021	159.291	0.390	155	
10	How difficult was it for you to wash dishes?	1.017	0.916	142.853	0.749	155	
11	How difficult was it for you to wash your hair?	0.874	0.899	140.233	0.796	155	
12	How difficult was it for you to tie shoelaces/knots?	0.754	0.657	102.570	1.000	155	non-uniform DIF dominant hand
**Work Domain**							
1	Unable to work because of hand(s)/wrist(s)?	1.214	1.254	189.349	0.016	150	
2	Shorten your work day because of hand(s)/wrist(s)?	0.747	0.690	104.213	0.998	150	
3	Have to take it easy at your work because of your hand(s)/wrist(s)?	0.627	0.612	92.453	1.000	150	
4	Accomplish less in your work because of hand(s)/wrist(s)?	0.587	0.573	86.491	1.000	150	uniform DIF education level
5	Take longer to do the tasks because of hand(s)/wrist(s)?	0.830	0.782	118.123	0.974	150	
**Pain Domain**							
1	How often did you have pain in your hand(s)/wrist(s)?	0.785	0.744	101.209	0.987	135	
2	Please describe the pain you had in your hand(s)/wrist(s)	0.807	0.808	109.877	0.945	135	
3	How often did the pain in your hand(s)/wrist(s) interfere with your sleep?	0.625	0.657	89.284	0.999	135	
4	How often did the pain in your hand(s)/wrist(s) interfere with your daily activities (such as eating or bathing)?	0.847	0.842	114.571	0.898	135	
5	How often did the pain in your hand(s)/wrist(s) make you unhappy?	1.027	1.142	155.354	0.111	135	non-uniform DIF location
**Aesthetics Domain**							
1	I am satisfied with the appearance (look) of my hand.	1.234	1.178	179.069	0.059	151	
2	The appearance (look) of my hand sometimes made me uncomfortable in public.	0.714	0.666	101.215	0.999	151	
3	The appearance (look) of my hand made me depressed.	0.558	0.541	82.266	1.000	151	
4	The appearance (look) of my hand interfered with my normal social activities.	0.823	0.794	120.644	0.967	151	non-uniform DIF education level
**Satisfaction Domain**							
1	How satisfied are you with the overall function of your hand?	0.646	0.598	89.654	1.000	149	
2	How satisfied are you with the motion of the fingers in your hand?	0.717	0.675	101.321	0.999	149	
3	How satisfied are you with the motion of your wrist?	1.269	1.392	208.748	0.001	149	item misfit
4	How satisfied are you with the strength of your hand?	0.841	0.832	124.744	0.927	149	
5	How satisfied are you with the pain level of your hand?	0.716	0.682	102.354	0.999	149	
6	How satisfied are you with the sensation (feeling) of your hand?	0.965	0.937	140.532	0.678	149	

a. Outfit MNSQ = outlier-sensitive mean square statistic

b. Infit MNSQ = information-weighted mean square statistic

c. DOF = degrees of freedom

d. DIF = differential-item-functioning

**Table 4:** Overall domain characteristics.

Domain	Cronbach’s α	Martin-Lӧf Test^b^
**Function**	0.872	0.997
**ADL**	0.949	1
**Work**	0.902	0.226
**Pain**	0.858	0.808
**Aesthetics**^a^	0.762	n/a
**Satisfaction**	0.870	0.97

a. The Aesthetics domain only contained 3 items after removing item 4 and could not be tested for unidimensionality.

b. The Martin-Lӧf test represents a p-value with significance of p<0.05. Significant results indicate multidimensionality of the domain.

### ADLs

**Fig 2:**
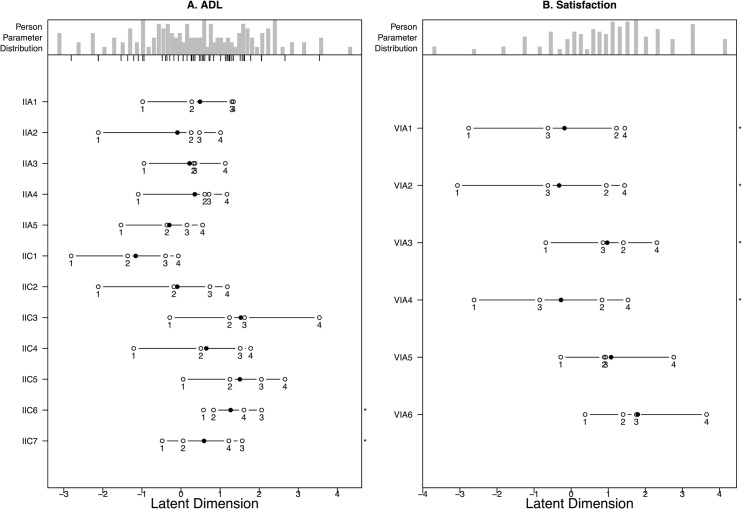
Disordered thresholds for ADLs (2a, left) and Satisfaction (2b, right).

Two items (wash your hair and tie shoelaces/knots) had disordered thresholds with difficulty discriminating between “very difficult” and “moderately difficult” (Fig 2A).

Additionally, five items (turn a door knob, pick up a coin, hold a glass of water, turn a key in a lock, and tie shoelaces/knots) had non-uniform DIF for dominant hand.

After collapsing thresholds (Fig 1B) and removing all items with non-uniform DIF, the remaining items showed excellent fit to the Rasch model (Table 3). No additional DIF was observed for dominant hand, education level, or location and the items were well-targeted to the study population. ADL had high internal consistency (Cronbach’s α = 0.94), was unidimensional according to the Martin-Lӧf test (p = 1), and had no unusual patterns in the residuals indicating no response dependency (Table 4). After removing a few items and collapsing disordered thresholds, ADLs showed excellent fit to the Rasch model.

### Work

No disordered thresholds were observed (Fig 1C) and all items fit well to the Rasch model (Table 3). One item “How often did you accomplish less in your work because of problems with your hand/wrist” showed uniform DIF for education level that was resolved after independently calibrating the domain for each subgroup. Work was well targeted and no other DIF was observed for hand dominance, education level or location. Work showed high internal consistency with a Cronbach’s α of 0.90, was unidimensional according to the Martin-Lӧf test (p = 0.23), and did not show any response dependency (Table 4). Overall, Work fit well to the Rasch model.

### Pain

**Fig 3:**
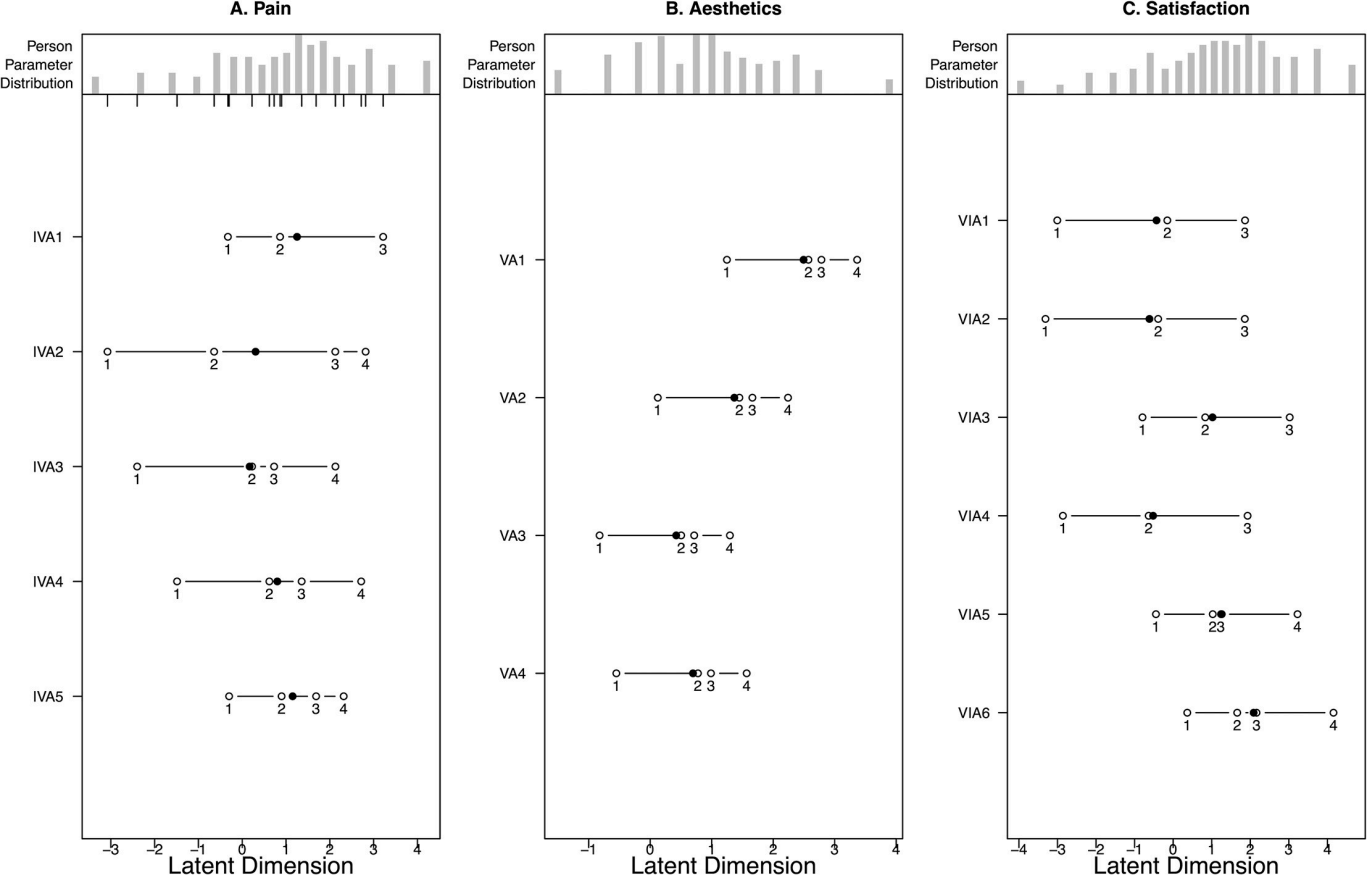
Person-Item Map and Threshold Ordering for Pain (3a, left), Aesthetics (3b, middle), and Satisfaction after collapsing disordered thresholds (3c, right).

No disordered thresholds were observed (Fig 3A) and all items fit the Rasch model well (Table 3). Pain was well targeted, did not show DIF for hand dominance or education level, and had excellent internal consistency with a Cronbach’s alpha of 0.85 (Table 4). Item 5 (How often did the pain in your hand make you unhappy?) showed non-uniform DIF for location and was removed from analysis. Pain was unidimensional according to the Martin-Lӧf test (p = 0.81) and no unusual patterns were observed in the residuals indicating local independence. Overall, after removing item 5, Pain showed excellent fit to the Rasch model.

### Aesthetics

No disordered thresholds were observed (Fig 3B) and all items fit well (Table 3). Non-uniform DIF for education level was observed for item 4 (“The appearance (look) of my hand sometimes made me uncomfortable in public”). No DIF was observed for dominant hand. Aesthetics had good internal consistency with a Cronbach’s α of 0.76 and did not show any unusual patterns in the residuals indicating no response dependency (Table 4). After removing item 4, there were not enough items to test for unidimensionality. Overall, Aesthetics showed adequate fit to the Rasch model, but could not be tested for unidimensionality following removal of item 4.

### Satisfaction

Disordered thresholds were observed for items 1–4 (satisfaction with overall function of your hand, motion of the fingers in your hand, motion of your wrist, and strength of your hand) with participants having difficulty distinguishing between “somewhat dissatisfied” and “neither satisfied nor dissatisfied” (Fig 2B). Additionally, item 3 (motion of your wrist) showed poor fit (p = 0.001) to the Rasch model.

Following removal of item 3 and collapsing of disordered thresholds (Fig 3C), all items had excellent fit to the Rasch model (Table 3). No DIF was observed for hand dominance or education level and Satisfaction had high internal consistency with a Cronbach’s α of 0.87 (Table 4). Satisfaction was unidimensional according to the Martin-Lӧf test (p = 0.97) and no response dependency was observed in the residuals. Overall, after removing item 3 and collapsing disordered thresholds, Satisfaction showed excellent fit to the Rasch model.

## Discussion

Our results show that after removing several misfitting items and collapsing disordered thresholds, the MHQ fits well to the Rasch model. Although the MHQ was derived from CTT, Rasch analysis showed that the MHQ has excellent adaptability as an interval-level instrument after making certain adjustments. Because the MHQ was derived from CTT, it is expected that some adjustments will be needed to convert an ordinal scale into an interval level measurement. However, all domains demonstrated local independence, internal consistency, and excellent targeting to the SARA cohort. Additionally, two domains (Function and Work) satisfied all Rasch criteria. The remaining four domains (ADLs, Pain, Aesthetics, and Satisfaction) required some adjustments before fitting the Rasch model.

In ADLs, disordered thresholds were observed for two items (wash your hair and tie shoelaces/knots) with participants unable to distinguish between “very difficult” and “moderately difficult.” Collapsing response options such that “very difficult” and “moderately difficult” are combined into one category addressed this problem, while maintaining a consistent ordering of thresholds across the continuum. Additionally, five items (turn a door knob, pick up a coin, hold a glass of water, turn a key in a lock, and tie shoelaces/knots) showed non-uniform DIF when the dominant hand was injured. Previous studies on patients with RA have demonstrated that individuals with increased mechanical stress experience increased damage to their dominant hand, which could result in the abnormal response pattern observed in ADLs [32, 33]. However, ADLs showed no DIF for education level indicating acceptable construct validity across other demographic variables. In Pain, non-uniform DIF for location was observed for item 5 (How often did the pain in your hand make you unhappy?). This may demonstrate that the association between pain and mood may vary across different cultures. No DIF was observed for hand dominance or education for the Pain domain.

In Aesthetics, item 4 (“The appearance (look) of my hand sometimes made me uncomfortable in public”) showed non-uniform DIF for education level, indicating inconsistencies in the way participants with similar levels of hand deformities answered this item based on their education level. This could result from varying life experiences that cause some individuals to experience more anxiety about the appearance of their hand in public settings. After removing item 4 from analysis, all items fit well with high internal consistency and excellent targeting, however, we could not test Aesthetics for unidimensionality because there were too few items remaining in the domain. These results suggest that including an additional item in Aesthetics may help to more accurately assess the association between hand appearance and RA outcomes.

Finally, Satisfaction had disordered thresholds in four of the six items with participants having difficulty discriminating between “neither satisfied nor dissatisfied” and “somewhat dissatisfied.” This could be the result of too many response options or ambiguity in the phrasing of these response categories. However, collapsing these responses addressed this limitation and maintained the latent structure of the domain. Additionally, item 3 (how satisfied are you with the motion of your wrist?) showed misfit to the Rasch model. This item could misfit because some participants who rely more on their wrist for daily activities may be less satisfied with their wrist motion than others with less reliance on their hand/wrist.

After these modifications, all domains satisfied Rasch criteria with the exception of Aesthetics which could not be tested for unidimensionality. Within the domains of the MHQ, the Function and Work domain scores showed ideal fit to the Rasch model. The remaining 4 domains required some adjustments before fitting the Rasch model. These results suggest that the Function and Work domains may be more reliable and valid than the other domains for interpreting results from MHQ administration. As a result, averaging all domains of the MHQ to create a summary score may not be indicative of a patient’s true experience living with rheumatoid arthritis because each domain may not equally contribute to a patient’s overall assessment of their hand outcome. By identifying the domains that are most accurate for measuring outcomes in patients with RA, we suggest clinicians focus more on the interpretation of individual domain scores when assessing outcomes in patients with RA. Additionally, we hope future investigators will use Rasch analysis to investigate the psychometric properties of outcome instruments used in RA to observe if other instruments would also benefit from a domain-specific interpretation. These results can help personalize RA treatment and optimize the way the MHQ is interpreted and applied in clinical practice.

Although certain domains fit the Rasch model better than others, we currently do not recommend making modifications to the MHQ. This is the first Rasch analysis performed on the MHQ in a RA population with MCP joint deformities and certain items that misfit in this cohort may fit well in other RA populations. By removing certain items in the MHQ after a single analysis, we may reduce the content validity of the overall assessment tool. Repeated Rasch analyses in other RA populations are needed to identify specific items that consistently misfit the Rasch model. If future analyses demonstrate similar item misfit, it presents an opportunity to develop an alternative version of the MHQ that is more specific to the RA population. Because the MHQ was developed as a generalized PROM that can be used to evaluate a variety of upper extremity musculoskeletal conditions, there may be certain items/domains that are more important in certain conditions. For example, our study demonstrates that the Function and Work domains may be more accurate for assessing outcomes in a RA population. However, other domains in the MHQ such as ADLs or Satisfaction could be more important in traumatic injuries such as finger amputations or distal radius fractures. Therefore, making significant changes to the MHQ could affect its ability to be used for other upper extremity conditions. Repeated Rasch analyses of the MHQ in other RA populations, however, could lead to the development of an alternative, RA-specific version of the MHQ that is more accurate for patients with RA.

Similarly, several items in the MHQ required collapsing of disordered thresholds to fit the Rasch model. This suggests that there are too many response options in the MHQ and that fewer response options may retain the properties of the questionnaire while maintaining its validity and reliability. Although certain categories required collapsing of thresholds, it does not limit the current version of the MHQ, which can still discriminate among patients with different levels of hand performance. In its current state, we recommend clinicians continue to use the complete MHQ for analyzing patients with RA. After more Rasch analyses are performed, an alternative, RA-specific version of the MHQ can be developed and a conversion system will be created that can easily convert scores between the old MHQ and the newer more RA-specific version of the MHQ.

Overall, the few adjustments required to satisfy Rasch criteria are not large enough to justify a modification to the MHQ at this time, and more studies are needed to investigate if similar misfit occurs in other populations before attempting to develop an RA-specific alternative to the MHQ. Moreover, these additional studies can provide clarity in how much each domain contributes to the MHQ to develop a summary score that weights each domain appropriately in a RA population.

One limitation is that the majority of enrolled SARA participants were white females, and these results may not translate across other racial and gender characteristics. Additionally, this Rasch analysis was performed using baseline data at single point in time, and does not analyze the psychometric properties of the MHQ following RA treatment over time. Finally, this study is specific to patients with MCP joint deformities and patients with RA with other hand/wrist problems may not respond the same way.

After removing misfitting items and collapsing disordered thresholds, all domains in the MHQ except for Aesthetics showed high validity and reliability in patients with RA with MCP joint deformities. The Aesthetics domain fulfilled all criteria except for unidimensionality. A domain-specific interpretation of the MHQ may provide more insight into RA outcomes than an overall summary score. Additionally, more studies are needed to identify items in the MHQ that consistently misfit in a RA cohort before any adjustments are considered in the MHQ.

## Supporting information

S1 Data SARA validation raw data. (XLSX)
